# The effects of short-term alpha-ketoisocaproic acid supplementation on exercise performance: a randomized controlled trial

**DOI:** 10.1186/1550-2783-4-2

**Published:** 2007-07-13

**Authors:** Joshua F Yarrow, Jeffrey J Parr, Lesley J White, Paul A Borsa, Bruce R Stevens

**Affiliations:** 1 University of Florida, Department of Applied Physiology & Kinesiology, Center for Exercise Science, Gainesville, FL 32611, USA; 2 Malcolm Randall VA Medical Center, GRECC, Gainesville, FL 32608, USA; 3 University of Florida College of Medicine, Department of Physiology and Functional Genomics, Gainesville, FL 32610, USA

## Abstract

**Background:**

This study examined the efficacy of short-term alpha-ketoisocaproic acid (KIC) monotherapy supplementation immediately prior to moderate- and high-intensity single bout exercise performance.

**Methods:**

Thirteen resistance trained men (22.8 ± 2.5 years; 81.6 ± 12.6 kg) participated in a prospective, randomized, double blind, placebo controlled crossover experiment. Each subject completed one familiarization and four experimental trials with either 1.5 g or 9.0 g of either KIC or isocaloric placebo control (CONT), following an overnight fast. During the experimental trials, subjects consumed the supplement regimen and then completed leg and chest press repetitions to failure and 30 s of repeated maximal vertical jumping (VJ) on a force plate.

**Results:**

In this treatment regimen, no significant differences (p > 0.05) were observed between dosages or conditions for leg press (low CONT = 19.8 ± 0.4 SEM, low KIC = 21.0 ± 0.5, high CONT = 20.1 ± 0.3, high KIC = 22.4 ± 0.6) or chest press (low CONT = 18.1 ± 0.2, low KIC = 18.5 ± 0.3, high CONT = 17.8 ± 0.3, high KIC = 18.0 ± 0.3) repetitions to failure. Additionally, no significant differences were observed for peak or mean VJ performance (low CONT = 34.6 ± 2.2 cm and 28.6 ± 1.8 cm; low KIC = 35.6 ± 2.0 cm and 29.4 ± 1.6 cm; high CONT = 35.7 ± 2.1 cm and 29.4 ± 1.7 cm; high KIC = 34.8 ± 2.3 cm and 28.3 ± 1.7 cm), respectively.

**Conclusion:**

Based on our results, we conclude that acute KIC ingestion by itself with no other ergogenic supplement, immediately prior to exercise, did not alter moderate- nor high-intensity single-bout exercise performance in young resistance-trained males. This study addressed single-dose single-bout performance events; the efficacy of KIC monotherapy supplementation on repeated high-intensity exercise bouts and long-term exercise training remains unknown.

## Background

More than 70% of athletes report using nutritional supplements to improve health and/or sports performance, [1] however, few supplements have shown ergogenic potential under controlled experimental conditions [19]. Alpha-ketoisocaproic acid (KIC), the ketoacid of leucine, has been shown to enhance high intensity exercise performance when consumed in combination with glycine and L-arginine (GAKIC) [6,26], and reduce exercise induced muscle damage and preserve skeletal muscle force production when ingested with β-hydroxy-β-methylbutyrate (HMB) [27] However, the possibility that lone KIC supplementation augments exercise performance has not been previously explored.

With moderate- and high-intensity exercise, factors such as total energy supply or local muscle fatigue may limit maximal anaerobic power [8] through reduced force output in skeletal muscle [9]. Theoretically, supplemental KIC may enhance exercise performance by 1) promoting total muscle energy supply, [5,11,28] 2) attenuating NH_3 _accumulation, [15,21] 3) and/or reducing exercise induced muscle damage [27]. For example, KIC administration is purported to spare glucose utilization by skeletal muscle, possibly by inhibiting glycogen deposition and/or the pyruvate dehydrogenase enzyme complex in skeletal muscle [5,7]. Further, the liver is capable of converting KIC to ketone bodies [11,28], thus increasing the potential energy supply during exercise [2]. Additionally, KIC is readily aminated to leucine, via leucine dehydrogenase and/or branched chain amino acid transferase at the expense of NH_3_, in a variety of tissues including skeletal muscle, [14,15,21] which may ultimately decrease fatigue and preserve skeletal muscle force production during intense exercise [3].

Although KIC supplementation has the potential to augment moderate- and high-intensity exercise performance, an effective dosage of KIC has not been determined. We have previously reported that serum KIC concentrations increase 150–300% following ingestion of 1.5 g–9.0 g KIC [29]. Therefore, the purpose of this study was to test the efficacy of supplementing both a low (1.5 g) and high (9.0 g) dosage of KIC immediately prior to single bout exercise performance. We hypothesized that supplemental KIC would improve both moderate- and high-intensity muscle performance, compared to placebo, and that a higher dose would have more pronounced results than a lower dose of KIC.

## Methods

### Subjects

Thirteen males (22.8 ± 2.5 years; 81.6 ± 12.6 kg; 16.7% body fat) volunteered from the general University population. The number of participants exceeded that indicated by an *a priori *power analysis (β = 0.80, α = 0.05). To be included, subjects must have participated in upper and lower body resistance training a minimum of three days week-1 for the previous two months and been accustomed to one-repetition maximum (1RM) testing. Subjects were excluded if they were a competitive athlete, had taken any supplement intended to improve muscle performance during the previous month, or self-reported a diagnosis of diabetes mellitus, aminoacidurias, maple syrup urine disease, renal failure, muscle wasting, hypertension, abdominal radiotherapy, intestinal resection, or acute illness. All study participants signed a written informed consent, approved by the University Institutional Review Board.

### Study Design

**Figure 1:**
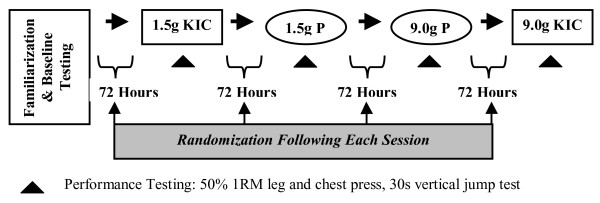


The overall design of this study was a prospective, randomized, double blind, placebo controlled, crossover scheme. Each subject performed a familiarization trial and four experimental trials. Prior to each experimental trial, KIC (Pharmaceutical Ingredients Ltd., Dayton, N.J.) or an isocaloric placebo (sucrose) was orally consumed in 500 mg capsules. Each subject participated in two low dose sessions and two high dose sessions in a completely randomized order (Figure 1). During the low dose conditions, the supplement was consumed 25 minutes prior to exercise testing. Whereas in the high dose conditions the supplement was consumed in two equal doses (45 minutes and 25 minutes prior to testing), in order to offset the possibility of any intestinal distress that might be associated with high dose bolus amino acid supplementation. The dosing schedule and specific dosages selected for this study (1.5 g and 9.0 g) were chosen based on previous work from our laboratory indicating that serum KIC concentrations increase ~150–300% within 45 minutes of ingesting 1.5 g, 3.0 g, and 9.0 g KIC [29].

In our study, no more than two trials were performed per week, with a minimum of 72 hours separating sessions. Participants were instructed to continue their normal resistance-training program; however, a minimum of 48 hours layoff from resistance exercise was required prior to each experimental trial. No additional attempt was made to control outside activity other than to request they perform their normal daily activities. Additionally, each experimental trial was conducted following a 10-hour overnight fast to account for the possible influence of energy intake on exercise performance.

### Familiarization Session

During the first laboratory visit, anthropometric measurements (body mass and body composition) were collected. Body mass was measured on a calibrated medical scale. Body composition was determined using the 7-site skinfold method [12]. Additionally, a 1RM was performed on leg press and chest press exercise machines (Med-X Corporation, Ocala, FL) according to standard protocol [4]. The 1RM results were used to individualize load assignments for subsequent leg press and chest press tests at 50% 1RM. Following 1RM testing, subjects performed three familiarization tests, a 1) 50% 1RM leg press test, 2) 50% 1RM chest press test, and 3) 30 s repeated maximal vertical jump (VJ) test on a triaxial force plate (Bertec Corporation, Columbus, OH).

### Testing Protocol

During experimental trials, subjects performed the 50% 1RM leg press and chest press test, with pre-determined loads, and the 30 s repeated VJ test. Each test was separated by five minutes to offset the possible impact of fatigue on performance results [20,24]. Continuous verbal encouragement was given during all performance testing. A description of each test follows.

### 50% 1RM Tests

For the 50% 1RM test, participants performed chest press and leg press repetitions to failure at a speed of 15 repetitions min^-1^, to the cadence of a metronome [18]. The number of repetitions completed for each test was used for statistical analyses.

### 30 s Repeated Vertical Jump Test

A standardized warm-up of five maximal jumps followed by a 1-minute rest period preceded VJ testing. For the VJ test, subjects were instructed to jump as high as possible for 30 seconds with self-selected jumping form, while minimizing ground contact [10]. Ground reaction force was recorded using the DATAPAC 2000 analog data acquisition, processing, and analysis system (Run Technologies, Laguana Hills, CA). Peak and mean VJ height, total work, [10] and work decrement [13] were used for statistical analyses.

### Blinding Protocol

Ketoisocaproic acid and placebo (isocaloric sucrose) were packed in identical 500 mg gelatin capsules and a blinding code was placed on each supplement. Supplement capsules for each treatment dose were consumed all at once with sugar-free sweetened and flavored water.

### Statistical Analyses

The SPSS 11.0 statistical package was used for all statistical analyses. Separate 2 (condition) × 2 (dose) repeated measures ANOVAs were run to determine differences in the outcome measures (total number of repetitions for the 50% 1RM tests and peak VJ height, mean VJ height, total work, and work decrement for the 30 s VJ test) in this investigation. An alpha level of p ≤ 0.05 was selected as the criteria for statistical significance.

## Results

During the study, two subjects reported gastrointestinal distress during both the high dose sucrose placebo and high dose KIC trials. The performance of these two subjects was analyzed separately and appeared consistent with the remaining subjects. Separate data analyses were performed with and without (n = 11) these subjects and the outcomes were consistent; therefore, the data from all subjects (n = 13) was used for statistical analyses. No further side-effects were reported for either dose of KIC or placebo.

### 50% 1RM Tests

**Figure 2:**
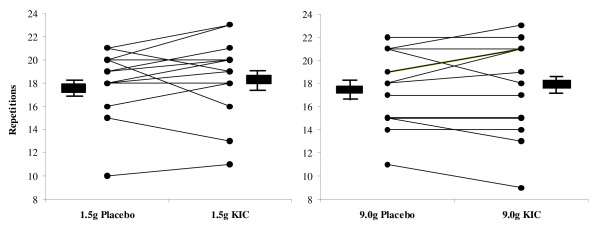


Results from the 50% 1RM tests are presented in Figures 2 (Chest Press) and 3 (Leg Press). The graphs depict individual performance scores and group mean performance scores ± standard error (SE). No significant differences between conditions (KIC vs. placebo) or dose (high dose vs. low dose) for number of repetitions performed on either the 50% 1RM leg press or chest press test were observed (p > 0.05).

**Figure 3:**
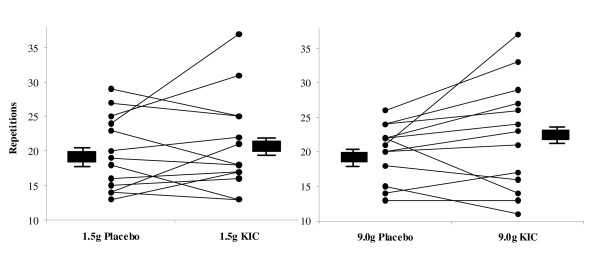


### 30 s Vertical Jump Test

**Figure 4:**
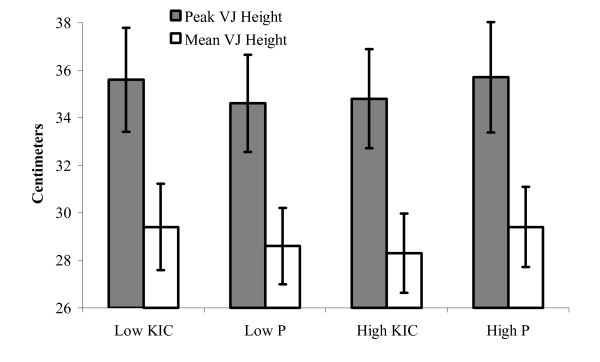


Results from the 30 s VJ test are presented in Figures 4 (Peak and Mean VJ) and 5 (Work Decrement); graphs depict group mean performance scores ± SE. The VJ results are based on the performance of 10 subjects because data on three subjects was lost due to equipment failure. The peak and mean VJ values (cm) ± SE for the 30 s VJ test were 34.6 ± 2.2 and 28.6 ± 1.8 (low placebo), 35.6 ± 2.0 and 29.4 ± 1.6 (low KIC), 35.7 ± 2.1 and 29.4 ± 1.7 (high placebo), and 34.8 ± 2.3 and 28.3 ± 1.7 (high KIC), respectively. Work was reduced by ~18–19% similarly across all groups. The total work values (Joules) ± SE were 5968 ± 360 (low placebo), 6170 ± 410 (low KIC), 6097 ± 369 (high placebo), and 6067 ± 365 (high KIC). No significant differences between conditions or doses were observed for peak or mean VJ height, work decrement, or total work (p > 0.05).

**Figure 5:**
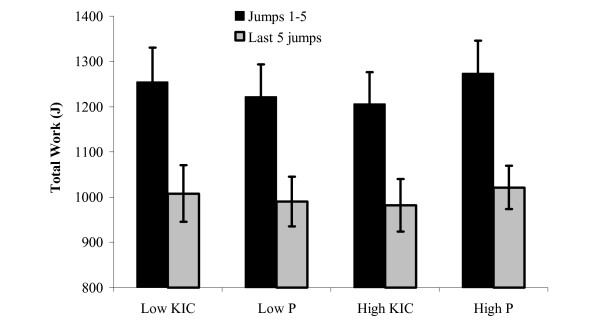


## Discussion

We designed our study to determine the efficacy of consuming either a low (1.5 g) or high (9.0 g) dose of KIC immediately prior to moderate- and high-intensity single bout exercise performance. Contrary to our hypothesis, KIC ingestion did not improve exercise performance, at either dose. Although there are no published reports documenting the efficacy of lone KIC supplementation on moderate- or high-intensity exercise performance, previous studies have shown that 3.2 g KIC given in combination with 6.0 g glycine and 2.0 g L-arginine (GAKIC) enhanced high-intensity exercise performance [6,26].

The inconsistency of our findings with previous GAKIC studies [6,26] may, be due in-part to the differences in exercise protocols. For example, Buford and Koch [6] observed a delay in fatigue between *repeated *high-intensity 10 s maximal cycling bouts, performed after GAKIC ingestion. Additionally, Stevens et al. [26] reported that GAKIC enhanced isokinetic peak torque and total work, and delayed both intra- and inter-set fatigue during a *repeated *35-repetitions (90° sec^-1^) maximal isokinetic knee extension protocol. Further, van Someren et al. [27] reported that KIC + HMB reduced skeletal muscle damage following *repeated *bouts of resistance exercise. In contrast to previous studies, [6,26,27] we examined the efficacy of administering lone KIC prior to single bouts of exercise and observed no alterations in exercise performance attributable to KIC. Our results suggest that repeated high-intensity exercise bouts may be necessary for the prospective ergogenic effects of KIC to become apparent, as KIC potentially 1) reduces skeletal muscle NH_3 _concentrations, [21] 2) alters anaerobic energetics, [5,28] and/or 3) attenuates exercise induced muscle damage [27]. Future research evaluating the effects of KIC administration prior to *repeated *high-intensity exercise bouts may assist in delineating the ergogenic potential of KIC.

Alternatively, our results may suggest that the previously reported ergogenic effects of GAKIC are due to the inclusion of glycine and/or L-arginine in the GAKIC formulation. Diets supplemented with 1% glycine and 2% L-arginine have been shown to increase intramuscular creatine phosphate concentrations in laboratory animals [16], although, these results have not been verified in humans. Additionally, orally ingested L-arginine has been shown to improve exercise performance in cardiovascular disease patients [17,23], possibly by upregulating nitric oxide dependent skeletal muscle vasodilation [22]. Schaefer and Piquard [25] have also reported that a 3 g arginine infusion reduced both blood lactate and NH_3 _peak concentrations during cardiovascular exercise, suggesting that L-arginine has the potential to augment exercise performance in healthy individuals. To our knowledge, no previous research has compared the metabolic responses to KIC, glycine, and/or L-arginine supplementation prior to high-intensity exercise.

## Conclusion

In theory, short-term KIC supplementation may acutely enhance exercise performance through a variety of mechanisms. However, our results demonstrate that ingestion of either a low (1.5 g) or high (9.0 g) dose of monotherapy KIC prior to exercise does not alter moderate- or high-intensity exercise performance. The efficacy of KIC monotherapy supplementation prior to repeated high-intensity exercise bouts and long-term exercise training remains unknown.

## Competing interests

The authors (JFY, JJP, LJW, and PAB) declare that they have no competing interests. BRS serves as a consultant to Iovate Health Sciences Research Inc, and receives royalties assigned to GAKIC. All the authors in this study independently collected, analyzed, and interpreted the results, while Iovate had no role in data collection, analysis, interpretation, nor manuscript preparation in any manner.

## Authors' contributions

JFY participated in design of the study, collected data, analyzed statistics, and drafted the manuscript. JJP collected data and helped to draft the manuscript. LJW and PAB participated in design of the study and helped to draft the manuscript. BRS conceived of the study, participated in design of the study, analyzed statistics, and helped to draft the manuscript. All authors read and approved the final manuscript.
